# Bridging the Gap: Do Patient-Reported Outcome Measures Reflect Objective Knee Function After Cartilage Repair?

**DOI:** 10.3390/jcm14227895

**Published:** 2025-11-07

**Authors:** Tizian Heinz, Niklas Wegerich, Sebastian Frischholz, Ioannis Stratos, Konstantin Horas, Stephan Reppenhagen, Maximilian Rudert, Manuel Weißenberger

**Affiliations:** 1 Department of Orthopaedic Surgery, Koenig-Ludwig-Haus, University of Wuerzburg, 97074 Wuerzburg, Germany; 2 Frankfurt Centre for Bone Health and Endocrinology, 60313 Frankfurt, Germany

**Keywords:** PROM, IKDC, objective knee score, correlation, German Cartilage Registry (KnorpelRegister DGOU), cartilage repair

## Abstract

**Background/Objectives**: Focal cartilage defects of the knee are a common cause of pain and functional impairment. While several patient-reported outcome measures (PROMs) and objective scores have been developed to assess clinical knee status and functional impairment, the correlation between subjective PROMs and objective clinical findings after cartilage repair surgery remains unclear. A better understanding of this relationship could enhance the interpretation of registry data and improve clinical decision-making. **Methods**: This study analyzed 52 patients from the German Cartilage Registry (KnorpelRegister DGOU) who underwent cartilage repair surgery of the knee at a single orthopedic university center in Germany. All patients were re-evaluated in a standardized follow-up examination. PROMs from either the registry or the follow-up examination and objective findings, summarized using a modified objective International Cartilage Repair Society [ICRS] score, derived from the International Knee Documentation Committee (IKDC) 2000 knee examination form, were correlated using Spearman’s rank correlation coefficient. **Results**: Moderate and statistically significant negative correlations were observed between the objective ICRS score and Knee Injury and Osteoarthritis Outcome Score (KOOS) Symptoms (ρ = −0.420, *p* = 0.005), KOOS Quality of Life (QoL) (ρ = −0.377, *p* = 0.013), and the subjective IKDC score (ρ = −0.305, *p* = 0.028) The subjective IKDC score (IKDC–Subjective Knee Form) was also moderately and significantly correlated with the objective ICRS score (ρ = –0.305, *p* = 0.028). Other KOOS subscales (Pain, Activities of Daily Living (ADL), Sport) did not show statistically significant correlations with the objective ICRS score. **Conclusions**: PROMs provide valuable insights into patients’ perceived outcomes after cartilage repair surgery, but do not fully reflect objective functional recovery, underlining the importance of combining them with clinical assessments. Level of Evidence: III.

## 1. Introduction

Focal cartilage defects of the knee are a common cause of pain and limit both function and quality of life [1]. Therapy for these defects remains challenging, due to their limited capacity for self-repair; therefore, they represent an increased risk for progression of an early onset osteoarthritis and the coexistence of several different treatment options (e.g., autologous chondrocyte transplantation [ACT], osteochondral transplantation [OCT], debridement), with varying levels of success [2,3,4,5,6,7]. To evaluate treatment success, both subjective patient-reported outcome measures (PROMs) and objective clinical assessments are used [8,9,10]. PROMs such as the subjective International Knee Documentation Committee (IKDC) score and the Knee Injury and Osteoarthritis Outcome Score (KOOS) provide information about the patient’s subjective perception of pain, functional impairment, and quality of life [9,11,12]. In addition, functional testing in standardized tests, such as the single leg hop test for distance or knee extension strength tests, are performed and play a role in evaluating the stability, pathologies and performance of the knee joint, and are therefore part of the overall assessment of treatment success [13]. Although PROMs capture the patient’s subjective perception, their items are often directly related to structural and functional deficits that can be objectively quantified. Reported pain, swelling, feeling of instability or difficulties in walking, stair climbing, sitting and sport activities usually indicate underlying biomechanical deficits such as reduced muscle strength, limited range of motion or ligamentous instability. From a theoretical perspective, PROMs can therefore be seen as a translator of physiological capacity into the patient’s experience of symptoms and daily activities, and can be used to indirectly assess objective function. However, the extent to which subjective PROMs truly reflect objective knee function after cartilage repair remains insufficiently investigated, with inconsistent evidence among published studies. Therefore, the aim of this study was to analyze the relationship between PROMs (KOOS subscales and subjective IKDC) and objective clinical findings, as summarized in the objective International Cartilage Repair Society (ICRS) score, in patients following cartilage repair surgery of the knee.

## 2. Material and Methods

### 2.1. German Cartilage Registry (KnorpelRegister DGOU)

This study is based on data from the German Knee Cartilage Registry (KnorpelRegister DGOU). It is a nationwide multicenter registry, aiming to independently collect and analyze data on treatment outcomes and complications following surgical cartilage repair procedures [14]. The registry includes patients with cartilage defects of the knee, hip and ankle joints. Only patients who underwent knee surgery were included and analyzed in this study.

Both patient-specific characteristics and PROMs were collected prospectively. Patient specific data—including sex, age, smoker status, body mass index (BMI), and intraoperative findings such as defect size, defect localization, ICRS-grade of the defect, and surgical technique—were entered by the physician. PROMs included the subjective IKDC score, all five subscales of the Knee Osteoarthritis Outcome Score (KOOS: Symptoms, Pain, ADL, QoL, Sport), a Numeric Rating Scale for pain and a questionnaire assessing patients’ sports activity. Links to complete the scores and questionnaires were sent out via mail automatically at predefined intervals: preoperatively and at 6, 12, 24, 36, 60 and 120 months postoperatively. Each link remained active for 4 weeks after distribution.

### 2.2. Study Design and Data Collection

For this study, patients who underwent surgery at the single orthopedic university center in Germany and were simultaneously enrolled for the German Cartilage Registry (KnorpelRegister DGOU) for the knee, were called in for a standardized follow-up examination. During this, various clinical examinations were carried out in addition to a new assessment of the subjective IKDC score. The follow-up examinations were all conducted by a final-year medical student who had been specifically trained and supervised by a board-certified orthopedic surgeon between March 2024 and April 2025. The examiner was not blinded to the patients’ medical history or prior surgical procedures.

All examinations were performed both on the index and contralateral knee. To assess joint mobility, the active and passive range of motion (ROM) was recorded using a goniometer. The stability of the knee joint was evaluated via the anterior and posterior drawer test, Lachman test, pivot shift test, and valgus/varus stress tests at both 0° and 20° of knee flexion. Also, passive external rotation of the knee joint was assessed in 90° flexion and 30° flexion. Further clinical parameters included the recording of crepitation, whereby a distinction was made between crepitation in the anterior, medial and lateral part of the knee joint, dancing patella sign, Zohlens’ sign and McMurrays’ sign to look for possible meniscus pathology.

To assess functional parameters, an isometric strength test for knee extension and flexion in 90° flexion was performed with a factory calibrated handheld dynamometer (SagaSafe Model AMF-500, accuracy grade 1, Guangzhou Qianqiu Technology Co., CN, Guangzhou, China). Furthermore, the Timed Up and Go Test, 60 s single leg balance stance, single-leg hop test for distance and the timed 6 m single-leg hop test were performed. For each test, patients underwent two tries with the mean value being included for analysis.

### 2.3. IKDC and ICRS Score

The main objective of the study is to analyze the agreement between the PROMs from the register, the subjective IKDC score which was filled out by patients on the day of the examination with the clinical parameters recorded during the follow-up. To summarize the objective clinical parameters, we used the clinical categories of the objective IKDC 2000 Knee Examination Form. Since the radiological assessment required for the official IKDC final grade was not performed in our study, we could not formally calculate the IKDC final score. To avoid incorrect attribution, we therefore refer to this measure as “objective ICRS Score”, although it is otherwise identical in content grading rules and final score calculation to the objective IKDC score. The score includes five categories, namely effusion, range of motion deficit, ligament stability, compartment findings and the single-leg jump test for distance to assess functionality. Each category consists of one or more subcategories (e.g., Extension deficit or flexion deficit for the range of motion deficit category) which can all be classified into grades A (normal), B (nearly normal), C (abnormal), and D severely abnormal. The worst grade of all subcategories determines the overall grade for the category. The final score equals the worst grade among the effusion, ROM deficit, and ligament stability categories. All other categories must be included, and are documented in our data acquisition, but do not affect the final score [15,16]. For statistical purposes, these grades were recoded as numerical values from 1 (A) to 4 (D), with higher values indicating worse knee function.

### 2.4. Statistics

Data evaluation and statistical analysis were performed using IBM SPSS Statistics version 29.0.2.0 (IBM Corp., Armonk, NY, USA). Normal distribution was assessed using the Shapiro–Wilk test and histograms. Descriptive data are presented as mean ± standard deviation or percentages of the total. Correlations were calculated using the Spearman rank correlation coefficient because PROMs and the ICRS score were not distributed normally. Sensitivity analyses used rank-based partial correlations (Spearman) between PROMs and the objective ICRS score, adjusted for ICRS-grade. Wherever normal data distribution was violated, non-parametric testing was used. Missing data in individual KOOS subscales were handled by complete case analysis, meaning that patients with missing values in a specific subscale were excluded from the respective correlation analyses but remained included in all other analyses where data was available. Because this was an exploratory study, no formal correction for multiple testing was applied. Reported *p*-values should therefore be interpreted with caution. Furthermore, a priori power calculation was not conducted as this was an exploratory study.

## 3. Results

### 3.1. Patient and Defect Characteristics

Overall, 203 patients were registered in the German Cartilage Registry (KnorpelRegister DGOU) and underwent surgical procedures at single orthopedic university center as of 05/2025. All patients were contacted by phone or mail and were asked to participate in a follow-up examination for the study. Of these, 52 patients agreed, yielding a response rate of 25.6%.

The mean age at the time of surgery was 39.2 ± 11.9 years (range 21–65), and the mean BMI was 28.5 ± 5.8. Of the participants, 38 were male and 14 were female. Regarding smoking status, 9 participants were active smokers, 1 was a former smoker, and 42 were non-smokers. The mean follow-up time since surgery was 66.8 ± 31.2 months (range 17–107). In terms of cartilage lesions, 42 Patients (80.8%) had one, 9 patients (17.3%) had two defects and 1 patient (1.9%) had three defects. All defects were classified intraoperatively according to ICRS grades for cartilage lesions of the knee. The worst defect for each patient was ICRS grade 1 in 1 patient (1.9%), ICRS grade 2 in 8 patients (15.4%), ICRS grade 3 in 33 patients (63.5%) and ICRS grade 4 in 9 patients (17.3%). The etiology of the defect was traumatic in 28 patients (53.8%) and degenerative in 24 patients (46.2%). The mean clinical limb alignment, measured as the difference between intercondylar distance (varus) und intermalleolar distance (valgus), was −1.2 ± 2.3 cm (range −7.0 to +4.1 cm), with negative values indicating varus and positive values indicating valgus alignment. Overall, 23 patients (44.2%) presented with varus alignment, 24 patients (46.2%) with neutral alignment, and 5 patients (9.6%) with valgus alignment. For one patient, grading was not documented. Prior surgery on the index knee was absent in 34 patients (65.4%). A total of 15 patients (28.8%) had undergone a single surgery previously, 1 patient had undergone two, and 1 patient had undergone three prior surgeries. An overview of demographic and clinical baseline data is shown in Table 1.

### 3.2. Patient-Reported Outcomes and Correlations with the Objective ICRS Score

**Figure 1:**
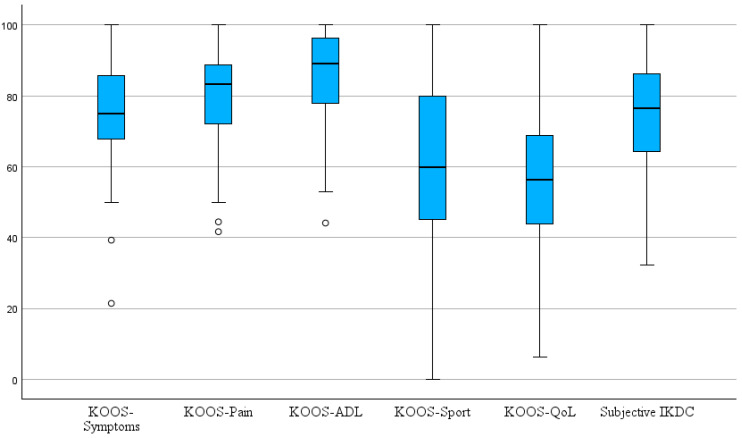
Latest available KOOS-subscale scores and subjective IKDC score. Boxplots showing the distribution of latest available KOOS-subscale scores and the subjective IKDC score across all patients. Circles represent statistical outliers.

**Figure 2:**
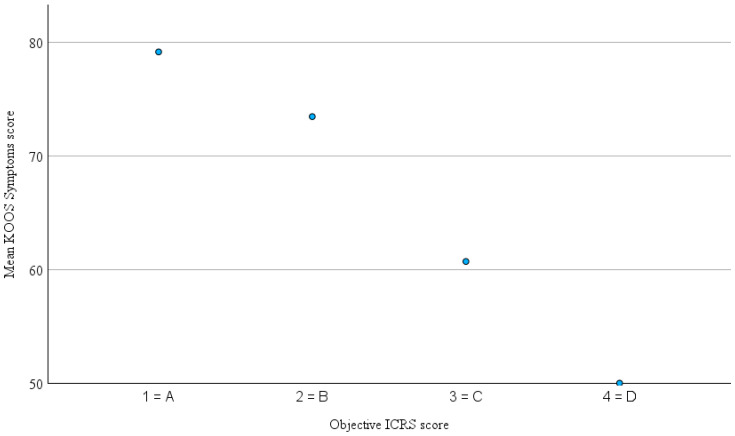
Mean KOOS–Symptoms score by objective ICRS score. Note: Higher ICRS scores indicate worse objective knee function.

**Figure 3:**
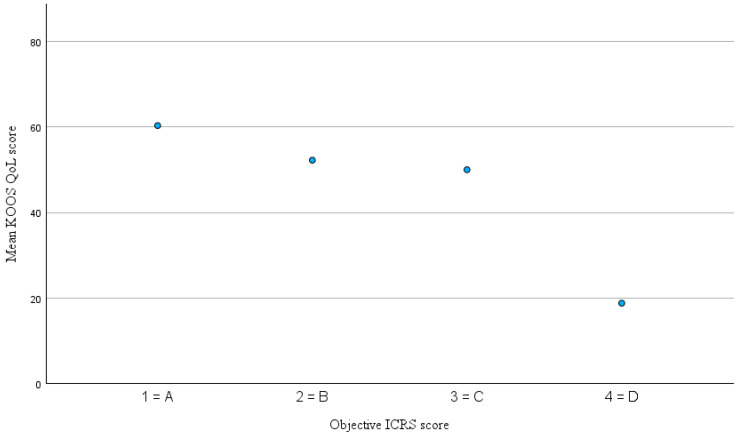
Mean KOOS–QoL score by objective ICRS score. Note: higher ICRS scores indicate worse objective knee function.

**Figure 4:**
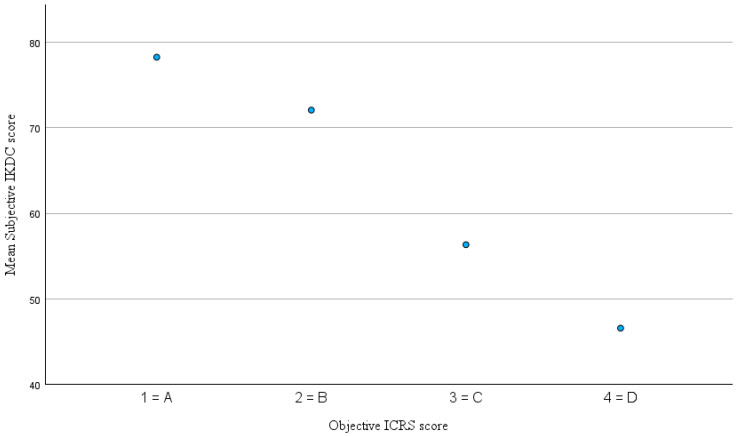
Mean subjective IKDC score by objective ICRS score. Note: higher ICRS scores indicate worse objective knee function.

The mean interval between the last collected KOOS, and the follow-up examination was 28.7 ± 19.4 month (range 3–85). The distribution of KOOS-subscale scores and subjective IKDC score is illustrated in Figure 1 and detailed results are presented in Table 2. The mean subjective IKDC score at follow-up was 74.6 ± 15.6 (range 32–100). Mean KOOSs for the five subscales were 79.1% ± 14.6% for KOOS–Pain, 75.5% ± 15.7% for KOOS–Symptoms, 55.5% ± 19.1% for KOOS–QoL, 59.2% ± 26.1% for KOOS–Sport and 85.7% ± 13.1% for KOOS–ADL. It is important to note that high scores for the KOOS subscales and the subjective IKDC score indicate better subjective knee function and less impairment, whereas higher objective ICRS scores indicate worse function (grade 1 = A = normal, grade 4 = grade D = severely abnormal). Spearman’s rank correlation analysis revealed a moderate correlation between the objective IKDC score and the KOOS–Symptoms subscale (ρ = −0.420, *p* = 0.005) as well as the KOOS QoL subscale (ρ = −0.377, *p* = 0.013). The corresponding scatterplots are shown in Figure 2 and Figure 3. Correlations with the remaining KOOS subscales were not statistically significant (KOOS–Pain: ρ = −0.268, *p* = 0.082; KOOS–ADL: ρ = −0.249, *p* = 0.107; KOOS–Sport: ρ = −0.198, *p* = 0.209). All calculated correlations are summarized in Table 3. Additionally, a significant negative correlation was observed between the objective ICRS score and the subjective IKDC score (ρ = −0.305, *p* = 0.028). This relationship is illustrated in Figure 4. For the correlations involving the objective and subjective IKDC scores, the sample size was *n* = 52, for the KOOS subscales, the sample size was *n* = 43. After adjusting for ICRS defect grade, the correlations between the objective ICRS score and PROMs were only slightly weakened and remain statistically significant for KOOS–Symptoms (partial Spearman ρ = −0.37, *p* = 0.019), KOOS–QoL (ρ = −0.32, *p* = 0.033), and the subjective IKDC score (ρ = −0.32, *p* = 0.043). Other KOOS subscales remained non-significant (all *p* > 0.1). Clinical coronal limb alignment did not show significant correlations with any of the PROMs (all *p* > 0.2) or with the objective ICRS score (ρ = 0.13, *p* = 0.37). This suggests that alignment was not a relevant confounding factor for the correlations between PROMs and der objective ICRS score. There were also no significant differences between traumatic and degenerative cases regarding PROMs or the objective ICRS score (all *p* > 0.3, Mann–Whitney U test). Also, patients with a single lesion and those with multiple lesions did not differ significantly in PROMs or in the objective ICRS score between these groups (all *p* > 0.17, Mann–Whitney U test).

## 4. Discussion

The present study revealed that PROMs—particularly the KOOS Symptoms and QoL subscales, as well as the subjective IKDC—show moderate correlations with the objective ICRS score. These observations indicate that selected PROM domains can to some extent mirror objective knee function following cartilage repair. While other KOOS subscales (Pain, ADL, Sport) did not reach statistical significance, these initial findings are nonetheless promising—especially given the limited existing research on this topic. The lack of significant correlations for KOOS Pain, ADL and Sport may be due to the relatively small sample size of our cohort. Notably, the correlation coefficients for these subscales were consistently negative, indicating that a true relationship may exist but could not be detected in our study due to insufficient power. Beyond methodological aspects, PROMs capture aspects of knee function that are influenced by factors other than objective functional knee status. The KOOS sport subscale, for example, may be severely influenced by the patient’s pre-injury activity level. Further studies with larger cohorts should further investigate why certain KOOS domains show weaker or inconsistent associations with objective clinical findings and investigate the influence of structural, functional and psychological factors on these subscales. They suggest that certain PROMs domains may indeed reflect objective functional outcomes, providing a valuable starting point for further exploration.

These findings are consistent with previous studies on patients undergoing anterior cruciate ligament (ACL) reconstruction and those with knee osteoarthritis, meniscal injuries, or cartilage repair [17,18,19,20]. In line with earlier research, weak to moderate correlations have been observed between PROMs and objective evaluations such as motion analysis, performance-based tests, and imaging scores like Magnetic Resonance Observation of Cartilage Repair Tissue (MOCART). For example, Ekanayake et al. reported that in 112 patients with end-stage knee osteoarthritis scheduled for total knee arthroplasty, KOOS–Junior scores showed only weak or no correlations with objective motion analysis parameters (ϱ = −0.01 for MICS alignment, ϱ = 0.33 for mobility, ϱ = 0.06 for total joint scores) [21]. Similarly, Oettl et al. found no significant associations between changes in PROMs (subjective IKDC and Core Outcome Measure Index [COMI]) and MRI-based MOCART or MOCART 2.0 scores in a cohort of 111 patients after cartilage repair. Only selected subgroups (e.g., AutoCart) demonstrated moderate correlations [22]. In line with these findings, Gauthier et al. reported that psychological readiness to return to sport, measured by the ACL-Return to Sport Injury Scale (ACL-RSI), did not correlate with parameters such as strength, performance in hop tests or knee stability in over 300 patients following ACL reconstruction [17]. Likewise, Logerstedt et al. demonstrated that low IKDC scores after ACL reconstruction were strongly associated with failing a functional return-to-activity test battery, whereas normal subjective IKDC scores did not reliably predict successful performance [18]. Taken together, these results support the view that PROMs and objective clinical data should be seen as complementary rather than interchangeable tools [21,22,23,24].

Furthermore, several conceptual limitations restrict the use of PROMs as a substitute for objective clinical measures. PROMs reflect the patient’s perception of symptoms, activity limitations and quality of life and may be influenced by psychological and contextual factors such as confidence in the joint or pre-injury activity levels. Also, day-to-day changes in PROMs are possible. Furthermore, PROMs are subject to ceiling and floor effects, with patients sometimes reporting high scores despite objective deficits or low scores despite high functionality. In contrast, objective assessments quantify biomechanical and physiological parameters and are not influenced by the patient’s subjective perception and may therefore detect impairments that patients no longer perceive. The fact that correlations are only weak to moderate should not be considered a methodological flaw but rather a reflection of these distinct yet complementary perspectives of joint health and functionality. A combined approach is likely to provide a more comprehensive and accurate evaluation, aiding clinical decision-making.

From a clinical perspective, the observed discordance between subjective and objective outcomes carries relevant implications for postoperative evaluation and rehabilitation management. Clinicians should be aware that good PROMs do not necessarily mirror restored objective function. This may be particularly true in patients who adapt their activity levels or expectations over time. Conversely, low PROM scores may not always be proof of structural failure but can be influenced by psychological, social or motivational factors.

A key implication of these findings concerns the use of PROMs in registry-based studies. Many national and international orthopedic registries, including the German Cartilage Registry (KnorpelRegister DGOU), rely heavily on PROMs due to their ease of collection and suitability for long-term follow-up with large sample sizes. However, the exclusive use of PROMs may overlook meaningful functional deficits. Therefore, datasets from large-scale registries, where objective assessments are not routinely available, clinicians should interpret PROM data with caution. Our results emphasize the limitations of PROM-only datasets and highlight the value of incorporating objective clinical evaluations—at least within structured, representative subcohorts—to enhance the validity of registry-based research.

While this study offers several strengths, including standardized in-person follow-up by a single examiner, it also has limitations. First, the relatively small sample size (*n* = 52) and low response rate (25%) represent potential sources of selection bias. It is possible that more health-conscious individuals or those with better surgical outcomes were more likely to participate, thereby limiting the generalizability of the results. Second, the temporal discrepancy between the registry-acquired KOOS scores and the follow-up examination introduces a time gap that may have affected the comparability between subjective and objective assessments. This interval may have allowed changes in patients’ clinical status between assessments, potentially distorting observed correlations. In addition, patients may adapt psychologically over time, modifying their perception of symptoms and functional limitations, which could influence correlations with later objective assessments. In contrast, the subjective IKDC score was obtained on the same day as the clinical follow-up, ensuring temporal alignment for this PROM and strengthening its comparability to clinical findings. Third, the lack of the radiological component required for the official IKDC final grade is a methodological limitation, and the examiner was not blinded to the patients’ medical history, introducing a possible observer bias. Although this ensured consistent examination procedures across all patients, no formal inter- or intra-rater reliability testing was performed. Finally, the heterogeneity of our cohort regarding surgical procedures, defect grades, prior surgeries, time since surgery, age, sex, and BMI presents both a limitation and a possible strength. Because of the limited sample size, no multivariable modeling or stratified analyses could be performed, which restricts the ability to account for potential confounders. Yet, this cohort represents the diversity of patients encountered in clinical practice, which may increase the pragmatic relevance of our findings.

Future studies with larger multicenter cohorts should apply multivariable and stratified analyses to explore the influence of surgical techniques, defect characteristics and patient-related factors on the relationship between PROMs and objective clinical findings.

## 5. Conclusions

PROMs show moderate correlation with objective functional assessment after cartilage repair, supporting their use in registries while highlighting the importance of complementary objective measures.

## Figures and Tables

**Table 1:** Patient and defect characteristics of the study population (*N* = 52).

Variable	Mean ± SD or *N* (%)
Age, years	39.2 ± 11.9
BMI, kg/m^2^	28.5 ± 5.8
Gender, male/female, *n*	38/14
Smoking status	Active: 9 (17.3%)
	Former: 1 (1.9%)
	Never: 42 (80.8%)
Follow-up time, months	66.8 ± 31.2
Clinical limb alignment, cm	Mean: −1.2 ± 2.3 (range −7.0 to +4.1)
Alignment categories	Varus: 23 (44.2%)
	Neutral: 24 (46.2%)
	Valgus: 5 (9.6%)
Etiology of defect	Traumatic: 28 (53.8%)
	Degenerative: 24 (46.2%)
Number of cartilage defects	1 defect: 42 (80.8%)
	2 defects: 9 (17.3%)
	3 defects: 1 (1.9%)
Worst ICRS grade (*n* = 51)	Grade 1: 1 (1.9%)
	Grade 2: 8 (15.4%)
	Grade 3: 33 (63.5%)
	Grade 4: 9 (17.3%)
Previous surgeries on index knee	None: 34 (65.4%)
	1: 15 (28.8%)
	2: 2 (3.8%)
	3: 1 (1.9%)

Notes. Values are presented as mean ± SD or *n* (%). Clinical limb alignment was measured as the difference between the intermalleolar distance (valgus) and the intercondylar distance (varus); negative values indicate varus and positive values valgus. Abbreviations: BMI, body mass index; ICRS, International Cartilage Repair Society; SD, standard deviation; *n*, number of patients.

**Table 2:** Patient-reported outcome measures (PROMs).

Outcome Measure	Mean ± SD	Range	*N*
Subjective IKDC	74.6 ± 15.6	32.2–100.0	52
KOOS–Symptoms	75.5 ± 15.7	21.4–100.0	43
KOOS–Pain	79.1 ± 14.6	41.7–100.0	43
KOOS–ADL	85.7 ± 13.1	44.1–100.0	43
KOOS–Sport	59.2 ± 26.1	0.0–100.0	42
KOOS–QoL	55.5 ± 19.1	6.3–100.0	43

Notes. Values are presented as mean ± standard deviation (SD). Higher scores indicate better knee function. Abbreviations: PROMs, patient-reported outcome measures; KOOS, Knee injury and Osteoarthritis Outcome Score; IKDC, International Knee Documentation Committee; ADL, Activities of Daily Living; QoL, Quality of Life.

**Table 3:** Spearman’s rank correlations between PROMs and the objective ICRS score (*n* = 52).

Variable Pair	Spearman’s ρ	*p*-Value	Interpretation
KOOS–Pain vs. objective ICRS	–0.268	0.082	weak, not significant
KOOS–Symptoms vs. objective ICRS	–0.420 *	0.005	moderate, significant
KOOS–QoL vs. objective ICRS	–0.377 *	0.013	moderate, significant
KOOS–ADL vs. objective ICRS	–0.249	0.107	weak, not significant
KOOS–Sport vs. objective ICRS	–0.198	0.209	weak, not significant
Subjective IKDC vs. objective ICRS	–0.305 *	0.028	moderate, significant

Notes. Spearman’s rank correlation coefficient (ρ) was used. Asterisks (*) indicate statistical significance at p < 0.05. Higher objective ICRS scores indicate worse clinical knee status. Interpretation of effect size: weak (ρ < 0.3), moderate (0.3 ≤ ρ ≤ 0.5), strong (ρ > 0.5). Abbreviations: PROMs, patient-reported outcome measures; KOOS, Knee injury and Osteoarthritis Outcome Score; ICRS, International Cartilage Repair Society; IKDC, International Knee Documentation Committee; ADL, Activities of Daily Living; QoL, Quality of Life. Bold Text in the table highlights significant correlations.

## Data Availability

The datasets generated and/or analyzed during the current study are available from the corresponding author on reasonable request.
